# Efficacy of second-time vacuum-assisted breast excision in the management of screen-detected high-risk breast lesions

**DOI:** 10.1186/bcr3698

**Published:** 2014-11-03

**Authors:** M Morgan, R Patel, W Teh

**Affiliations:** 1 Northwick Park Hospital, Harrow, UK; 2 North London Breast Screening, London, UK

## Introduction

Surgical excision of breast lesions is usually required for high-risk (B3) lesions or radiological pathological discordance. The risk of underestimation of malignancy is 45 to 50% for 14G core biopsy and 20 to 25% for vacuum-assisted biopsies (VAB).

## Methods

In our institution, all screen-detected microcalcifications or ultrasound occult lesions are subjected to 11G VAB. If a discordant or high-risk lesion (B3) - for example, atypical ductal hyperplasia (ADH), lobular neoplasia or papillary lesion - is diagnosed on the initial VAB, an 8G **vacuum-assisted breast excision (**VAE) may be performed following MDT discussion.

## Results

Between 2011 and 2013, 1,250 VAB were performed on 990 women. Eighty-three 8G VAEs were performed during this time, and of these 62.7% of lesions (52/83) were classified histologically as B2, 24.1% (20/83) remained B3, 2.4% (2/83) as B4, 7.2% (6/83) as B5a and 2.4% (2/83) as B5b. Following 8G VAE, 45 patients (54.2%) were discharged to screening, six patients (7.2%) underwent annual surveillance, diagnostic surgery was performed on 23 patients (27.7%) and therapeutic surgery on eight patients (9.6%).

## Conclusion

Our study demonstrates that 8G VAE after an initial B3 diagnosis on VAB can avoid diagnostic surgery and improve cancer detection. Surgery was not required in 51 of the 83 cases (61.4%) of B3 lesions found on initial 11G VAB. A total of 17 B3 lesions (20.5%) were upgraded after 8G VAE; nine by diagnostic surgery (12%) and eight of the 83 cases of B3 lesions (9.6%) by VAE to either B5a or B5b requiring definitive surgery. Lesions most likely to be upgraded to malignancy were lobular neoplasia and ADH.

